# Follitropin alfa biosimilars: unfounded doubts on the road to reproductive care

**DOI:** 10.1093/humrep/deaf143

**Published:** 2025-07-26

**Authors:** Fernando de Mora, Colin M Howles, Geoffrey Trew

**Affiliations:** Department of Pharmacology, Therapeutics and Toxicology, Universidad Autónoma de Barcelona, Barcelona, Spain; Honorary Fellow, Deanery of Biomedical Sciences, University of Edinburgh, Edinburgh, UK; ARIES Consulting Sàrl, Geneva, Switzerland; Institute of Reproductive Sciences, Oxford, UK

Dear Editors,

We have read with interest Kiose *et al.* (2025) comparing the clinical outcome of different follitropin preparations. In essence, the authors conclude that biosimilar medicines’-induced pregnancy rates are likely to be lower than original follitropin alfa. Such a statement challenges the science of biosimilarity behind the European Medicines Agency (EMA)’s regulation proven successful for over 110 biosimilar medicines (covering 25 active substances). Who is driving the wrong way?

Kiose *et al.* make conceptual mistakes captured in the sentence ‘…*two biosimilars…have been approved in the EU, Ovaleap^®^ and Bemfola^®^, based on Phase III clinical studies and by demonstrating non-inferiority …*’. Beyond the fact that the trials were powered for equivalence rather than non-inferiority, asserting that biosimilar medicines approval is ‘*based on Phase III trials*’ is misleading. The least sensitive approach to pick-up differences is a comparative clinical trial; hence, EMA’s principle that structural and functional comparability is the foundation of biosimilar medicines approval (Wolff-Holz *et al.*, 2019) Interestingly, Merck supports that same notion (Magnenat *et al.*, 2017) which also explains that Gonal-f^®^’s intrinsic heterogeneity, and differences between original follitropin alfa and beta, are not substantiated clinically. Accordingly, the British Medicines & Healthcare products Regulatory Agency asserted that efficacy trials are not an effective discriminating tool in biosimilar development (Bielsky *et al.*, 2020), in alignment with a recently issued EMA reflection paper (EMA, 2025). However, if conducted, EMA regulators remind us that a tailored clinical program partly aiming at a reduction of the amount of clinical data required for the development of biosimilar medicines is scientifically meaningful (Guillen *et al.*, 2023). While, for instance, the overall response rate is not the efficacy measure in advanced follicular lymphoma, it is the preferred endpoint for comparability. Similarly, EMA recommends follitropin-mediated ‘number of oocytes retrieved’ as the best endpoint to prove similarity. In an analysis of complex biosimilar medicines, EMA experts (Kirsch-Stefan *et al.*, 2023) called ‘*…into question the usefulness of comparative efficacy studies*’, to the extent that in some cases where the efficacy trials failed, biosimilarity was ultimately accepted.

Evidence argues against homogeneity in the meta-analysis from Kiose *et al.* (2025). The authors wrongly identify Follitrope^®^ as a biosimilar medicine. Follitrope^®^’s development was not meant to demonstrate biosimilarity to Gonal-f^®^ (LG Chem, 2024; Ministry of Food and Drug Safety of South Korea (MFDS), 2024). Furthermore, neither biosimilarity of Afolia^®^ with the USA Gonal-f^®^ RFF Redi-Ject™, has been accredited, nor data from trial NCT01687712 were used for approval of Afolia^®^ in Australia. Hence, including the Afolia^®^ trial in the meta-analysis is erroneous. It is to the role of regulatory authorities, not researchers, to qualify a product as a biosimilar medicine. Moreover, the World Health Organization (WHO) has expressed concerns over sub-standard medicines being approved by weak regulatory systems (WHO, 2024). Copy-versions, often named Non-Original Biologics (NOB), available in non-Stringent Regulatory Authority (SRA) jurisdictions, pose a significant problem for patients. Contrary to Ovaleap^®^ and Bemfola^®^, four of the products included in the meta-analysis have not been authorized by a WHO Listed Authority (WLA). WLA is granted to an agency that complies with all the relevant requirements for regulatory capability, as defined by a performance evaluation process. Neither the Russian authority (Primapur^®^), nor Iran’s (Cinnal-f^®^), China’s (QL 1012), or Argentina’s (Folitime^®^) are currently WLA, or, more importantly, had gained the WHO SRA status in force at the time of approval (WHO, 2017). Only access to the nonpublic dossier would reveal if the level of structural sameness to Gonal-f^®^ of those products was comparable to that required by EMA, but differences exceeding the acceptable limits may explain that none of the four products were registered in Europe or the USA (e.g. Primapur^®^, see Manzi *et al.*, 2022).

Mistakenly identifying products as biosimilar medicines represents a major flaw in Kiose *et al.* Expectedly, a pooled analysis of the data from the two EU-approved biosimilar medicines, Ovaleap^®^ and Bemfola^®^, found no significant difference in live births. This observation should have warranted greater consideration in Kiose *et al.* discussion. Accordingly, an EMA post-approval study demonstrated no significant clinical differences when comparing Ovaleap^®^ to Gonal-f^®^ (Kaplan *et al.*, 2021).

All in all, the European Union monitoring system had not identified any relevant difference in the efficacy or in the adverse effects between biosimilar medicines and their reference medicines (Correia-Pinheiro *et al.*, 2021). No biosimilar medicine has been withdrawn or suspended. Biosimilar FSH penetration has increased steadily in the EU from a 4% share of the follitropin alfa market in 2016, up to 46% in 2025. Furthermore, a co-author of this letter who is the Chief Clinical Officer of the TFP IVF group, reports that the replacement of Gonal-f^®^ by Ovaleap^®^ (>12 000 cycles) across eight UK clinics, has not revealed any performance deviation in key clinical indicators. Accordingly, the 2025 revised ESHRE’s draft guidelines (The Updated ESHRE Guideline on Ovarian Stimulation for IVF/ICSI, 2025) do not discriminate among different preparations of alfa rFSH, and of other gonadotrophins, for ovarian stimulation. If Kiose *et al.* are confident that, despite the serious flaws in their meta-analysis, their conclusion that biosimilar medicines of Gonal-f^®^ registered in highly regulated jurisdictions (Bemfola^®^ and Ovaleap^®^) exhibit a lower clinical efficacy is strongly supported, they should report it to the EU authorities. Otherwise, by concluding against the science of biosimilarity, the authors are contributing to spread misinformation and distrust in biosimilar medicines, and consequently to artefactually reducing access to biologics causing a high cost of opportunity for patients.
